# Mining the Gap: New Opportunities for the WHO Surgical Safety Checklist

**DOI:** 10.1002/wjs.70317

**Published:** 2026-03-20

**Authors:** Nathan Turley, Mary E. Brindle

**Affiliations:** ^1^ Department of Surgery Cumming School of Medicine University of Calgary Calgary Alberta Canada; ^2^ Ariadne Labs Harvard TH Chan School of Public Health Harvard University Boston Massachusetts USA

What clinicians say and believe they do and what they actually do can be very different stories. Such is the finding of Rennie and colleagues' latest research on the WHO Surgical Safety Checklist (SSC) and the gap between a Belgium‐based endovascular surgical team's observed and reported adherence to the SSC's Time‐Out section [1]. This study highlights often unappreciated deficits in performance and points the way for teams to target these deficits to enhance safety in a continuous fashion.

The SSC was created to help teams perform at their best‐providing reminders of key steps and encouraging critical conversations. The SSC's Time‐Out engages teams when all members are co‐located the moment before a procedure begins. This part of the checklist tends to have the greatest compliance and enhances the culture of the OR by reinforcing the team's commitment to their patient and each other. Despite the Time‐Out's importance, the authors report a gap between reported and actual checklist compliance. They reflect that these findings may be symptoms of habitual SSC use degrading into obedient tick‐boxing to meet administrative requirements; however, the gap is not seen as an empty void, but rather a space for SSC optimization.

Put into context, Rennie et al.’s study is an important contribution to the expanding body of knowledge on the SSC in three ways. One, the authors' findings highlight the potential of electronic SSCs by countering concern that teams' communication and collaboration could suffer when teams trade paper SSCs for digital ones. Two, they propose a strategy of continuous video surveillance for providing real‐time feedback on performance while minimizing the Hawthorne Effect, and are the first study to deploy the CheckPOINT observation tool outside the tool's creators. Three, their findings reiterate the vital role surgeon leaders play in promoting the SSC's use.

The study results have important implications for teams who are reconsidering the SSC's format. The authors collected data on 212 endovascular procedures where the Time‐Out was either done using paper (*n* = 70, 33%) or using an electronic SSC (*n* = 142, 67%). When two raters separately observed and rated the performances of the team's Time‐Outs, there were differences in checklist‐item adherence, with the electronic version performing worse, but they saw no significant difference between formats for the teams' communication effectiveness, attitude, or engagement in the SSC. This is a salient finding because it defies concerns we and others have held regarding the inclusion of the SSC in the EMR and the potential degradation of teams' interactions during performance of the SSC. This study supports findings of Perera et al. study developing the CHOISSE framework for implementing e‐checklists using a sociotechnical systems (STS) in which they found that OR communication improved among staff once e‐checklists were introduced [2]. Research on the impact of SSC modality on team dynamics remains limited, yet promising.

Rennie's study is also noteworthy because it combines two novel data‐collection tools to capture and assess the gap between the endovascular teams' reported and observed adherence to the SSC during its Time‐Out section. Audio and video performances of the Time‐Out were captured using the Operating Room Black Box (ORBB) [3], whereas the quality of these performances was scored with the Checklist Performance Observation for ImprovemeNT (CheckPOINT) tool [4]. The ORBB captures multimodal feedback including environmental data, and audio and video input from the OR for AI‐enhanced review. This technology creates new opportunities for quality improvement and research where surgical personnel need to be observed, but episodic in‐person evaluation is time‐consuming, prone to error, and limited to defined periods of data collection. In‐person collection also runs the risk of the Hawthorne Effect: the tendency for study participants to act and talk how they believe their observers want them to act [5]. Outside of research, there are embedded flaws in traditional collection processes used in administrative audits, where the individual reporting compliance is also part of the team completing the SSC and may have external pressure to report good compliance. Rennie and colleagues demonstrate that taking the human observer out of the equation and replacing them with something that gradually melts into the background allows for natural observations. The team's use of ORBB also allowed evaluation of video footage of SSC performance separate from the event‐achieving potentially greater reliability, objectivity, and consistency.

The review of > 200 elective endovascular procedures captured by the ORBB in this study was performed using the CheckPOINT observation tool. A relatively new tool, CheckPOINT was developed to provide a reliable method of capturing team performance of the SSC rather than simple compliance. It builds on the communications and teamwork framework established by Devcich et al. WHO Behaviourally Anchored Rating Scale (WHOBARS) [6], while including a measure of checklist adherence [4]. CheckPOINT provides a rating system of SSC performances along four domains using 7‐point Likert scales: checklist adherence, communication effectiveness, attitude, and engagement. Although the wording changes for each of these domains, the scores range from extremely negative (1/7) to extremely positive (7/7). Rennie et al.’s observers achieved good inter‐rater reliability and moderate intra‐rater reliability, demonstrating the tool's ease of use and raters' consistency in evaluating surgical teams' performances. Whereas Moyal et al.'s study involved an in‐person administration of CheckPOINT, Rennie et al. leveraged the ORBB, so they could score true‐to‐life performances of the Time‐Out. This study represents the first use of CheckPOINT outside of studies performed by the original authors, and the reliability and reflections generated by the results point to the potential value of this tool.

Finally, Rennie et al.'s study furthers the SSC canon by exposing the need for stronger surgical leadership and reflects how the hierarchical structure of the OR promotes behavioral patterns among staff. The authors found that the more procedural experience a surgeon has, the less likely they are to complete the SSC, which speaks to issues of checklist buy‐in and implementation, and how surgical leadership influences both of them [7, 8]. Surgical champions of the SSC can make or break its implementation and revitalization efforts. Modeling the SSC's performance and completion, surgeons set the tone on the SSC's prioritization and influence how others perceive the checklist's role and use.

Although the gap between observed and reported SSC adherence is problematic, Rennie and colleagues' study suggests that gaps in performance can be mined for opportunities. Technology is rapidly evolving in procedural spaces‐posing risks and opportunities that are being discovered in near real‐time. Digitization of the checklist has raised concerns that charting of compliance within an EMR could be prioritized over human interaction; however, Rennie and colleagues have shown that this does not need to be the case. Furthermore, the evolution of continuous data capture and monitoring provided by technologies like the Black Box and the integration of tools that explore team performance like CheckPOINT enhance our understanding of quality of care provided in procedural spaces.

Beyond illustrating how emerging technologies may enhance checklist performance, Rennie et al. anchor their current findings in established evidence; re‐emphasizing fundamental quality‐improvement principles that are gaining renewed and urgent significance. Surgeon leadership and psychological safety remain a core piece of checklist success; and harmful hierarchies, if not addressed, can weaken team performance. The SSC should be tailored for the context in which it is used, as failure to match the SSC with the team's needs will decrease compliance and team buy‐in. The SSC must therefore continue to evolve to keep pace with changes in clinical demands, changing teams, and evolving technologies to remain relevant. Rennie et al. provide a glimpse at a possible future for a digitally integrated checklist paired with a strategy that can target real‐time human performance to achieve a continuous evolution toward excellence.

## Author Contributions

**Nathan Turley:** writing – original draft, conceptualization, writing – review and editing. **Mary E. Brindle:** conceptualization, writing – review and editing.

## Funding

The authors have nothing to report.

## Conflicts of Interest

The authors declare no conflicts of interest.

## Data Availability

The authors have nothing to report.
